# Supplementary material for: Heterologous HSPC Transplantation Rescues Neuroinflammation and Ameliorates Peripheral Manifestations in the Mouse Model of Lysosomal Transmembrane Enzyme Deficiency, MPS IIIC
Source: Cells. 2024 May 20;13(10):877. doi: 10.3390/cells13100877 (PMC11120110; doi:10.3390/cells13100877)
Supplement: Supplementary file 1 [file cells-13-00877-s001.zip › cells-2969762-supplementary.pdf]

Supplementary materials

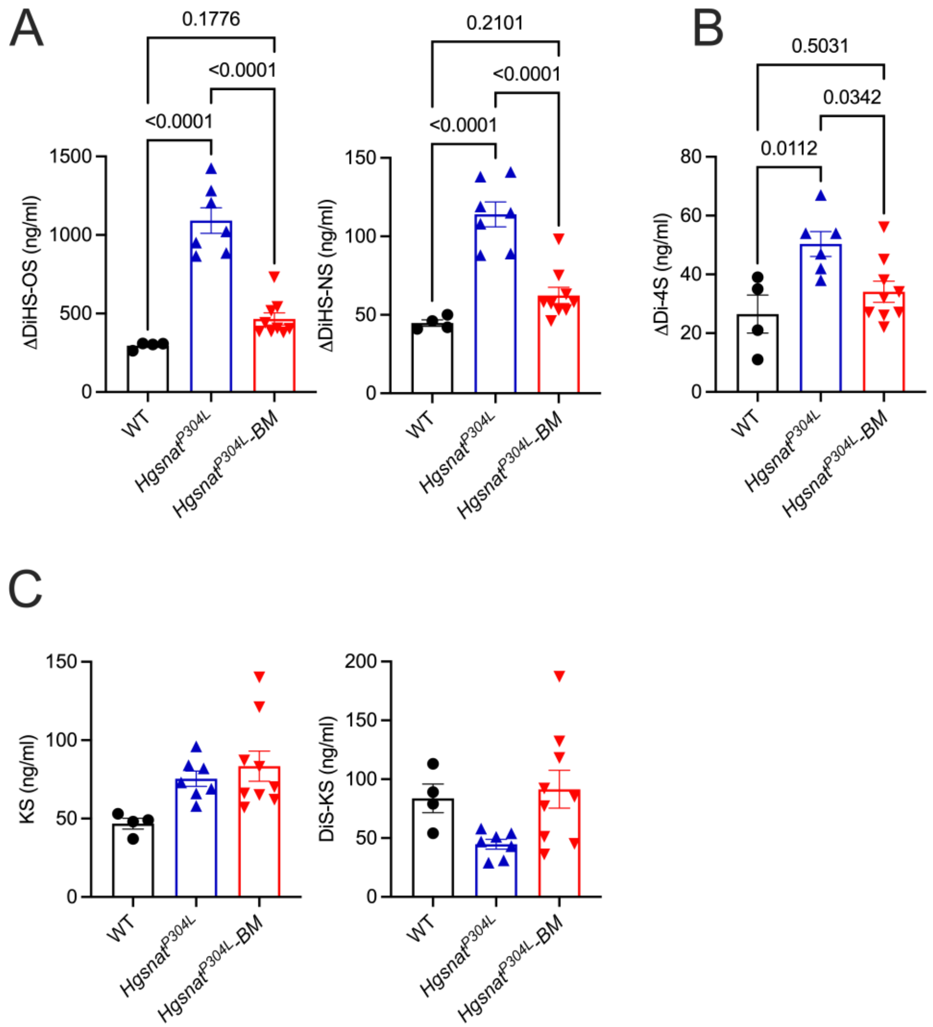

Figure S1. Levels of disaccharides produced by enzymatic digestion of HS, KS and DS in dry blood spots of mice 6 weeks after HSPC transplantation.

Levels of HS-derived O-sulfated (ΔDiHS-OS) and N-sulfated (ΔDiHS-NS) disaccharides **(A)** or dermatan sulfate-derived disaccharide (ΔDi-4S) **(B)** are increased in the DBS of untreated *Hgsnat*^{P304L} mice, while in the transplanted *Hgsnat*^{P304L} mice of the same age, the levels of all three disaccharides are not significantly different from the normal levels. **(C)** Levels of disaccharides produced by enzymatic digestion of mono (KS) and di-sulfated (DiS-KS) keratan sulfate are similar in DBS of WT, *Hgsnat*^{P304L} and transplanted *Hgsnat*^{P304L} mice. All graphs show individual data, means and SD of experiments performed with samples of 4-9 male and female mice per genotype per treatment. P values were calculated by one-way ANOVA with Tukey post hoc test.

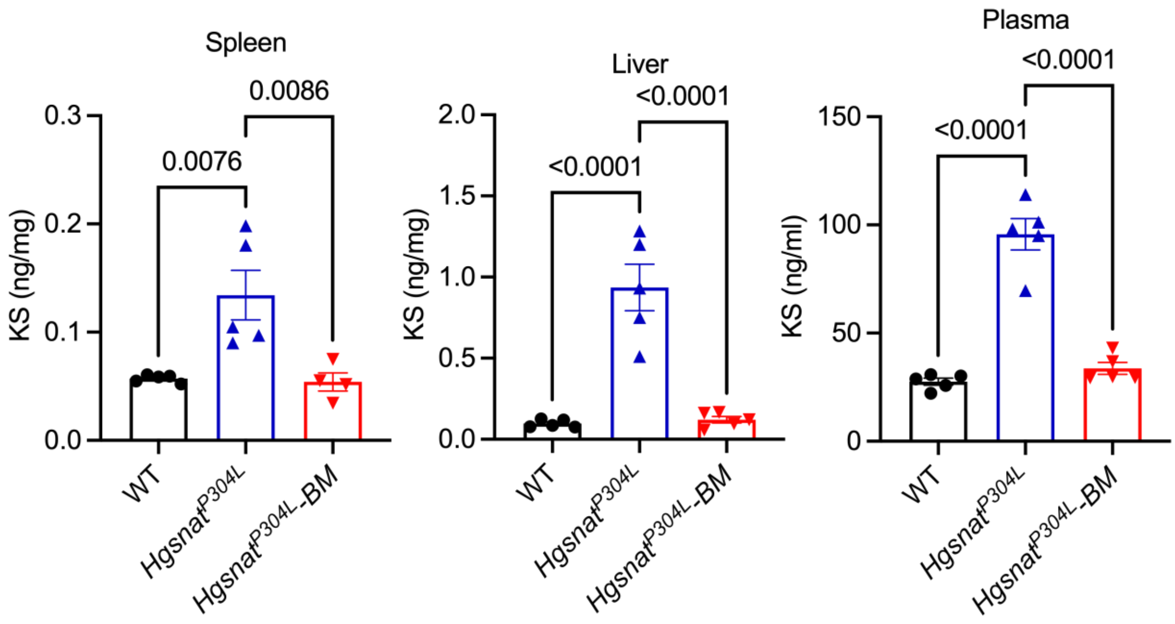

Figure S2. Levels of disaccharides produced by enzymatic digestion of mono-sulfated KS in blood plasma, liver and spleen of WT, *Hgsnat*^{P304L} and transplanted *Hgsnat*^{P304L} mice at the age of 8 months.

All graphs show individual data, means and SD of experiments performed using tissues from 5 male and female mice per genotype per treatment. P values were calculated by one-way ANOVA with Tukey post hoc test.

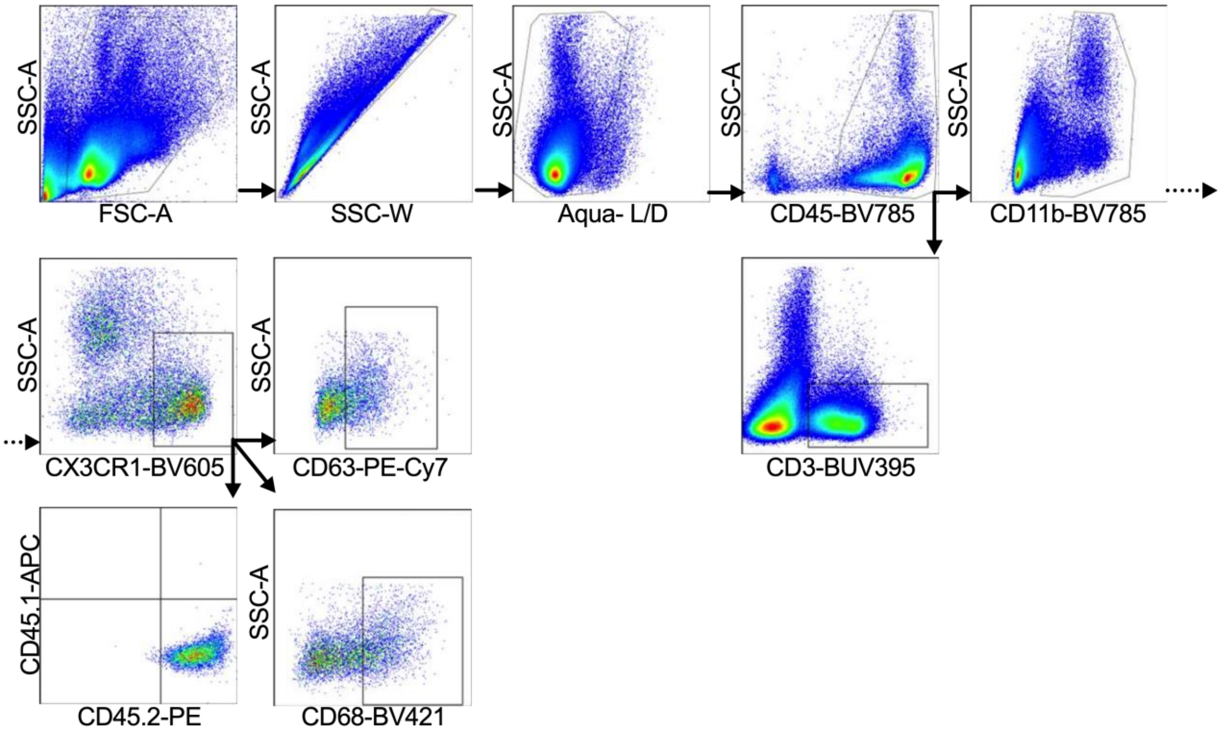

Figure S3. Gating strategy for the analysis of dissociated brain/spinal cord cells and splenocytes by flow cytometry.

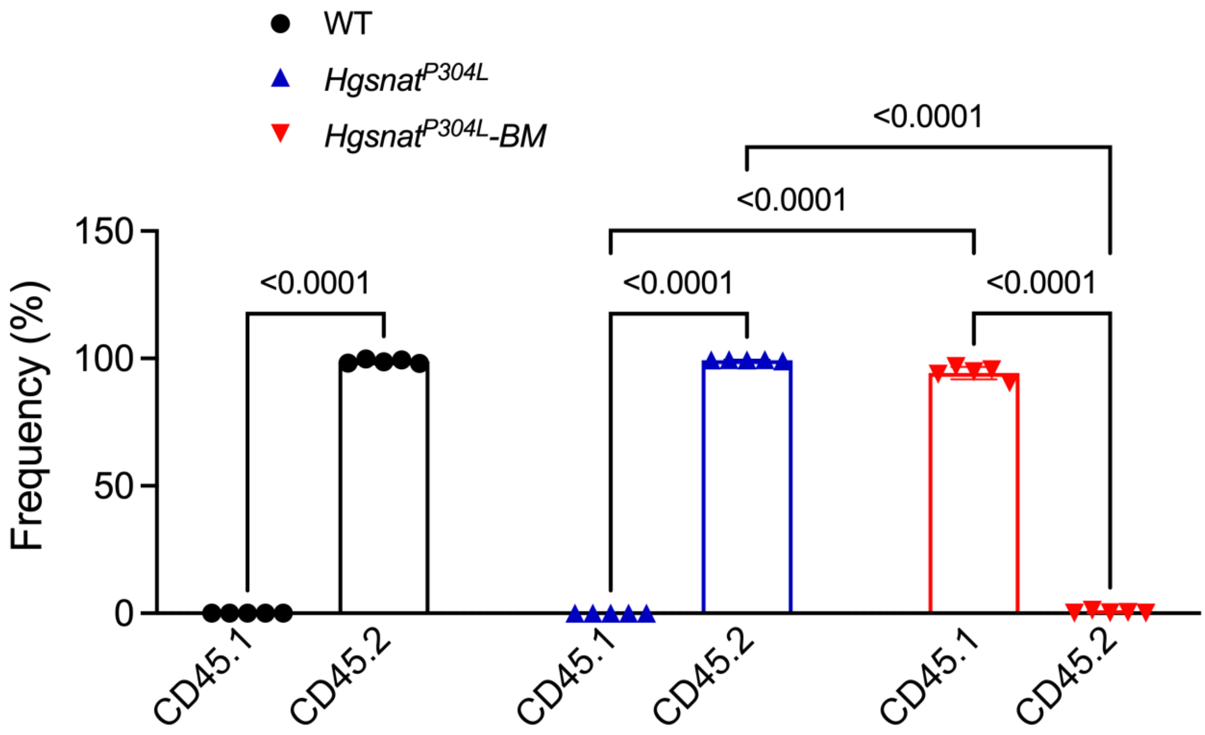

Figure S4. All CD45/CD11b/CX3CR1-positive macrophages in the spleen of transplanted *Hgsnat*^{P304L} mice show CD45.1 phenotype indicating that they are derived from transplanted HSPC.

Graphs show individual results, means and SD from experiments conducted with 5 mice per group. P values were calculated using one-way ANOVA test with Tukey post hoc test.

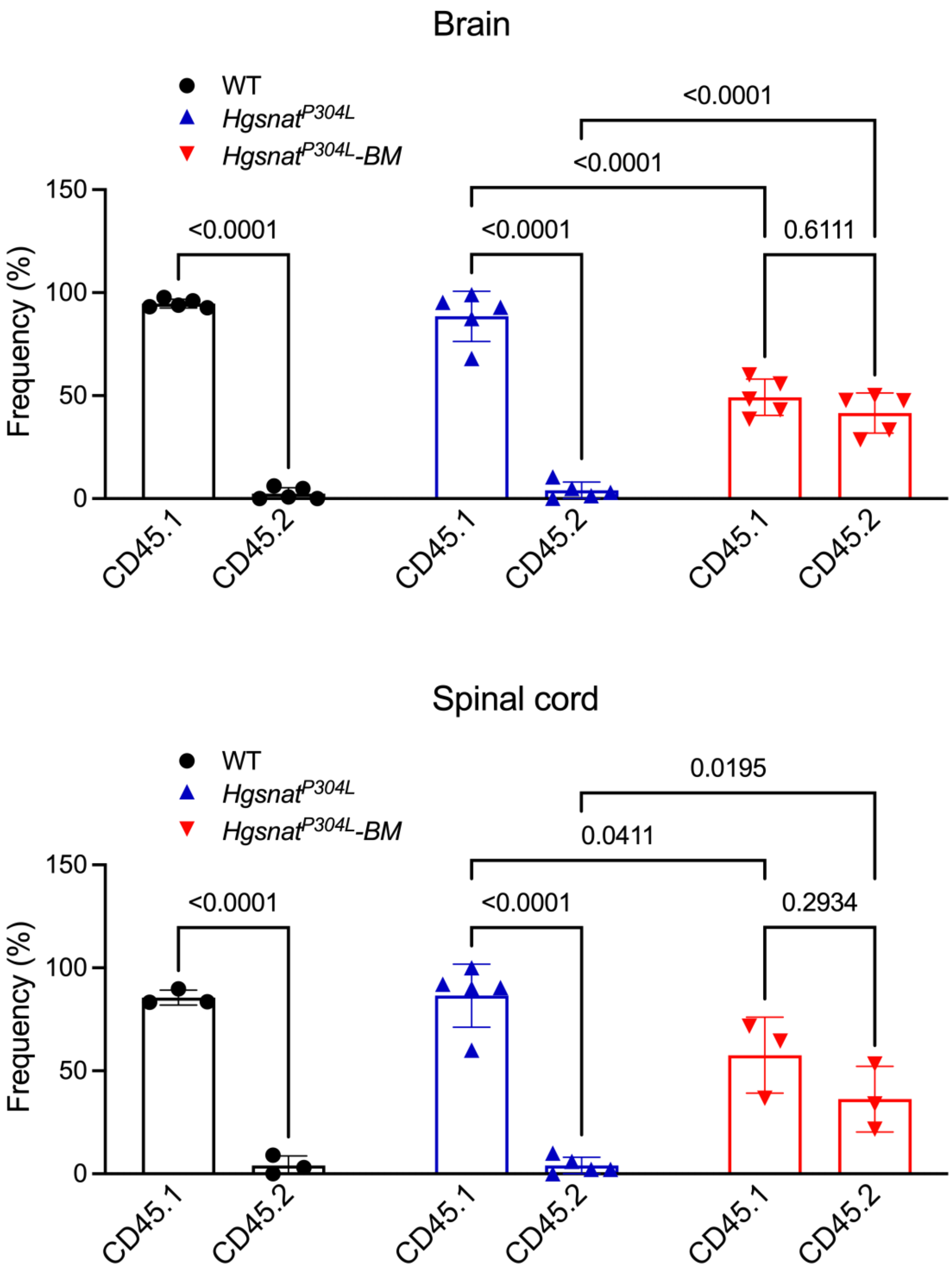

Figure S5. In both brain and spinal cord tissues approximately 50% of CD45/CD11b/CX3CR1-positive macrophages/microglia cells are CD45.1-positive and derived from transplanted HSPC.

Graphs show individual results, means and SD from experiments conducted with 3-5 mice per group. P values were calculated using one-way ANOVA test with Tukey post hoc test.

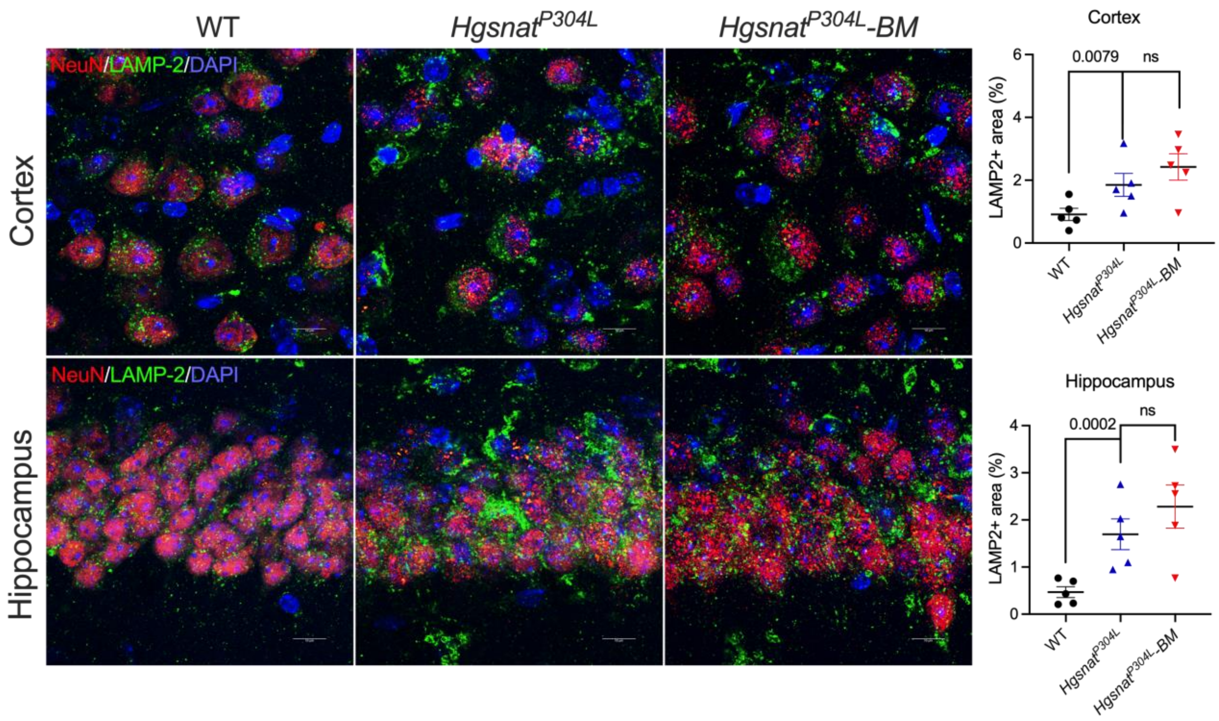

Fig. S6. Levels of LAMP-2-positive puncta are unchanged in cortical and hippocampal neurons of transplanted *Hgsnat*^{P304L} mice.

Panels show representative images of brain cortex (layers 4-5) and CA1 region of hippocampus of 8-month-old WT and treated or untreated *Hgsnat*^{P304L} mice labeled for LAMP-2 (green) and NeuN (red). DAPI was used as a nuclear counterstain. Scale bars equal 10 µm. Graphs show quantification of LAMP-2-positive areas in NeuN-positive cells with ImageJ software. All graphs show individual results, means and SD from experiments conducted with 5 mice (three panels per mouse) per genotype per treatment. P values were calculated using Nested one-way ANOVA test with Tukey post hoc test.

Supplementary Table 1. Mouse engraftment 6 weeks after transplantation

Body Weight before transplantation (g)	ID # /sex	CD45.1+ myeloid cells (%)	CD45.2+ myeloid cells (%)
26	11354 / F	83.7	15.1
30	11335 / M	84.5	14.2
30	11334 / M	88.2	10.6
33	11333 / M	82.0	16.8
23	11560 / F	88.0	9.9
24	11561 / F	87.6	12.3
24	11562 / F	86.8	12.3
30	11552 / M*	NA	NA
29	11553 / M	88.3	10.6
30	11554 / M	91.6	7.6
34	11556 / M**	NA	NA
29	11557 / M	94.1	4.9
24	11566 / F	90.8	7.9
26	11565 / F	91.4	7.0
25	11564 / F	93.2	6.4
24	11563 / F	91.3	8.0

* Died on the 4th week after transplantation
** Died on the 3d week after transplantation
